# Decreasing the Lag Between Result Availability and Decision-Making in the Emergency Department Using Push Notifications

**DOI:** 10.5811/westjem.2019.5.42749

**Published:** 2019-07-01

**Authors:** Christian Koziatek, Jordan Swartz, Eduardo Iturrate, Dina Levy-Lambert, Paul Testa

**Affiliations:** * New York University School of Medicine, Ronald O. Perelman Department of Emergency Medicine, New York City, New York; † New York University School of Medicine, Department of Medicine, New York City, New York

## Abstract

**Introduction:**

Emergency department (ED) patient care often hinges on the result of a diagnostic test. Frequently there is a lag time between a test result becoming available for review and physician decision-making or disposition based on that result. We implemented a system that electronically alerts ED providers when test results are available for review via a smartphone- and smartwatch-push notification. We hypothesized this would reduce the time from result to clinical decision-making.

**Methods:**

We retrospectively assessed the impact of the implementation of a push notification system at three EDs on time-to-disposition or time-to-follow-up order in six clinical scenarios of interest: chest radiograph (CXR) to disposition, basic metabolic panel (BMP) to disposition, urinalysis (UA) to disposition, respiratory pathogen panel (RPP) to disposition, hemoglobin (Hb) to blood transfusion order, and abnormal D-dimer to computed tomography pulmonary angiography (CTPA) order. All ED patients during a one-year period of push-notification availability were included in the study. The primary outcome was median time in each scenario from result availability to either disposition order or defined follow-up order. The secondary outcome was the overall usage rate of the opt-in push notification system by providers.

**Results:**

During the study period there were 6115 push notifications from 4183 ED encounters (2.7% of all encounters). Of the six clinical scenarios examined in this study, five were associated with a decrease in median time from test result availability to patient disposition or follow-up order when push notifications were employed: CXR to disposition, 80 minutes (interquartile range [IQR] 32–162 minutes) vs 56 minutes (IQR 18–141 minutes), difference 24 minutes (p<0.01); BMP to disposition, 128 minutes (IQR 62–225 minutes) vs 116 minutes (IQR 33–226 minutes), difference 12 minutes (p<0.01); UA to disposition, 105 minutes (IQR 43–200 minutes) vs 55 minutes (IQR 16–144 minutes), difference 50 minutes (p<0.01); RPP to disposition, 80 minutes (IQR 28–181 minutes) vs 37 minutes (IQR 10–116 minutes), difference 43 minutes (p<0.01); and D-dimer to CTPA, 14 minutes (IQR 6–30 minutes) vs 6 minutes (IQR 2.5–17.5 minutes), difference 8 minutes (p<0.01). The sixth scenario, Hb to blood transfusion (difference 19 minutes, p=0.73), did not meet statistical significance.

**Conclusion:**

Implementation of a push notification system for test result availability in the ED was associated with a decrease in lag time between test result and physician decision-making in the examined clinical scenarios. Push notifications were used in only a minority of ED patient encounters.

## INTRODUCTION

Decreasing emergency department (ED) length of stay and wait times is an ongoing effort in emergency medicine.^1^–^3^ ED crowding is a challenge, and increasing throughput is an objective for many institutions. Improvements in ED flow and crowding are associated with higher quality of care.^4^,^5^ Crowding is associated with higher stress levels among healthcare providers, longer wait times, increased boarding of admitted patients, and a higher rate of adverse events and poor outcomes.^6^,^7^ While many factors are associated with ED crowding, ED patients are often awaiting test results to affect a clinical disposition. This is an element of ED throughput that may be a target for quality improvement.^8^

Emergency physicians typically manage several patients simultaneously and make clinical decisions based on information that becomes serially available as tests result. Tracking the timing of resulting patient studies while caring for multiple patients is difficult and managed idiosyncratically by most physicians. Delays in responding to newly resulted test information (due to task-switching, interruptions, and other challenges of the ED clinical environment) likely impact patient throughput.^9^,^10^ Electronic systems that wirelessly alert providers about timed events have been shown to improve throughput in ED patients evaluated for chest pain.^11^,^12^ These alerts also increase the likelihood of the result reaching the provider and help avoid potential errors in communication of test results.^13^

At our institution we implemented the ability for providers to receive an electronic alert when the result of any selected test has been entered in the system. Providers are able to indicate their choice to receive such an alert at the point of order entry in the electronic health record (EHR). This alert is sent in the form of a push notification to handheld devices (smartphones and smartwatches) that have the mobile version of the EHR installed. The notification signals to the provider that a test result is available for viewing on either the smartphone or computer.

We chose to examine four commonly ordered tests in the ED to evaluate whether a push notification about the availability of these results reduced the time to a disposition decision being made about patients (discharge vs admission). Additionally, we examined two clinical scenarios to evaluate whether the time to ordering a follow-up intervention was reduced by the new alerting mechanism. The first scenario evaluated the time from a critically low hemoglobin result (<7 grams per deciliter) was entered into the system and a blood transfusion was ordered; the second scenario was the time from an abnormal D-dimer result to the time when a computed tomography pulmonary angiogram (CTPA) was ordered. These scenarios were chosen a priori by the study investigators as clinical decisions most clearly related to the result of a single preceding test result. Our hypothesis was that the new alerting system would reduce the lag time between result availability and physician decision-making.

**Population Health Research Capsule**

What do we already know about this issue?

Electronic health records can push notification of results to smartphones; this strategy has been shown to reduce time to disposition in chest pain patients.

What was the research question?

Does a push notification system decrease lag time to decision-making in several clinical scenarios of interest?

What was the major finding of the study?

Use of result push notifications was associated with decreased time to decision-making in several clinical scenarios.

How does this improve population health?

Push notifications are a strategy that busy emergency departments may consider to help address issues of crowding and improve throughput.

## METHODS

### Study Setting and Population

New York University (NYU) Langone Health is an integrated health network in New York City with three EDs that collectively evaluate 150,000 patients per year. NYU Tisch Hospital is a tertiary care academic medical center with approximately 75,000 visits per year, NYU Cobble Hill is a free-standing ED with approximately 24,000 visits per year, and NYU Brooklyn Hospital is a Level 1 trauma center with approximately 52,000 visits per year. We collected data on patients from July 1, 2017, when the push notification functionality was made available, through June 30, 2018. In that time period, 78 ED providers subscribed to at least one push notification (37 attending physicians, 24 resident physicians in emergency medicine, and 17 physician assistants).

### Study Design

This was a retrospective, multicenter study to evaluate a quality improvement initiative. ED providers were free to subscribe to push notifications on whatever studies they chose and on whichever patients they chose. Providers were notified of this new functionality via departmental email update. Any order not yet resulted after being placed could be selected for a result push notification. This included orders placed by nursing or any other provider. Providers were not blinded as blinding in this setting would have been infeasible. Providers were never prevented from accessing result data via the traditional log-in, computer-based EHR (Epic Systems Corporation, Verona, Wisconsin), even if they were using push notifications. EHR data was queried from the Epic Systems Clarity database with the use of Oracle SQL Developer (Oracle Corporation, Redwood City, California) and exported for data analysis; the queried data included encounter ID, notification type, notification time, order time, order-resulted time, and disposition time (defined as order to either admit or discharge the patient from the ED). The study was approved by the institutional review board of the NYU School of Medicine.

### Outcomes

The outcomes of interest in this study included the following: time from the result of a chest radiograph (CXR) being made available to the time of disposition; time from basic metabolic panel (BMP) result available to disposition; time from urinalysis (UA) result available to disposition; time from respiratory pathogen panel (RPP) result available to disposition; time from hemoglobin (Hb) result available to time blood transfusion was ordered; and time from D-dimer result available to time of CTPA order. Point-of-care laboratory tests (eg, troponin, lactate) – the results of which are communicated directly from the test performer (the nurse) to the test orderer (physician or physician assistant) – were excluded from analysis because the results of these tests are available prior to being entered in the EHR. We also excluded advanced imaging studies from analysis because providers are frequently informed of critical results by radiology telephone call prior to their being entered into the EHR.

### Data Analysis

Median time with interquartile ranges (IQR) is reported in each scenario, and we used the Mann Whitney (Wilcoxon) test for unpaired data to assess whether the difference in medians between the two groups was statistically significant, defined as two-tailed p-values < 0.05 (R Statistics, version 3.3.3). For each of the measured scenarios we constructed a clustered boxplot comparing the median times with IQRs in the push notification and no push notification groups; minimum/maximum value whiskers were not displayed for visual scaling purposes (Excel, Microsoft Corporation, Redmond, Washington).

## RESULTS

During the study period there were 152,574 ED encounters: 148,391 ED encounters without a push notification (no notifications cohort), and 4183 ED encounters with a push notification (notifications cohort). There were 6115 push notifications generated from the notifications cohort, comprised of 4102 distinct patients. The median age, admission rate, average Emergency Severity Index, and gender percentages for the two patient cohorts are presented in Table 1. Overall, 32% (78/241) of ED providers subscribed to at least one notification during the study period: 28% (37/136) of attending physicians, 38% (17/45) of physician assistants, and 53% (24/45) of resident physicians. Fifteen of the 78 providers (19.2%) accounted for 79.7% of the notifications. Providers received result notifications about 320 unique lab/imaging studies. Of the 320 studies, 37 (11.6%) accounted for 79.8% of the total. There were four lab or imaging tests on average ordered per encounter in the push notification cohort. Push notifications were employed in 2.7% of all ED encounters during the study period. The overall rate of push notification subscriptions rose slightly over the study period, from 2911 push notifications during the first six months to 3204 push notifications in the second six months.

**Figure 1:**
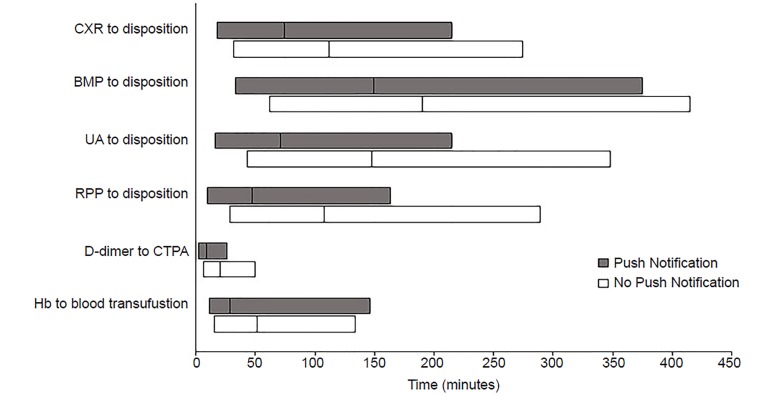
Boxplot of median minutes and interquartile ranges for time to disposition or time to follow-up order in each clinical scenario studied. *CXR*, chest radiograph; *BMP*, basic metabolic panel;* UA*, urinalysis; *RPP*, respiratory pathogen; *CTPA*, computed tomography pulmonary angiography;* Hb*, hemoglobin.

Of the six diagnostic tests we examined in this study, five were associated with a decrease in median time from test result availability to provider decision-making (Figure 1); the sixth scenario did not meet statistical significance. The largest improvements in median time from result to disposition were seen with the UA and RPP result notifications (50 and 43 minutes, respectively), whereas the improvement in time from result to disposition for the CXR and BMP results was more modest (24 and 12 minutes, respectively) (Table 2). In the follow-up order scenarios, the time from abnormal D-dimer to CTPA order was eight minutes faster in the push notification group; the time from critically low Hb result to blood transfusion was 19 minutes faster, but this finding was not statistically significant.

## DISCUSSION

This study’s findings demonstrate a correlation between employment of test-result push notifications to smart devices and improved patient care efficiency. Of the six diagnostic test types examined, all were associated with a decrease in lag time from result availability to the next clinical step – either patient disposition, or defined follow-up order. A larger magnitude of effect was observed for UA and RPP results than for CXR and BMP results. Both UA and RPP require specific collection (urine sample or nasopharyngeal swab), which commonly leads to delays, and both tests typically take longer to result than blood tests; result notifications may be more efficacious in the setting of tests that are slow to result. The improved time from D-dimer result to CTPA order was modest (eight minutes). In the setting of cascading delays in ED patients who first wait for blood test results and then imaging study results, even this small improvement in lag time may be relevant. Similarly, for the time from Hb result to blood transfusion, an improvement of 19 minutes would also be clinically meaningful, even though due to the small sample size of push notifications in this scenario (18 notifications) this finding did not meet statistical significance.

The result push-notification functionality described is inherent to the EHR used at our institution, and therefore any institution using this EHR can potentially use this functionality. At this time, however, there is no ability to default result push notifications for all providers, or for a given provider conditionally for a specific test (for example, to always push CXR result notifications). The requirement to manually opt-in each time a test is ordered may have limited the magnitude of effect and depressed the usage rate of the push notification functionality in our study. While the overall rate of push notification usage did rise slightly during the study period, and a large number of physicians and physician assistants used the push notification system at least once, there was a low overall percentage of patient encounters in which push notifications were employed by the provider (2.7% during the study period). A notably larger percentage of resident physicians opted to use the push notifications than attending physicians, which may be due to role-related workflows (residents primarily managing the patient flow) as well as age-related factors (younger resident physicians may be more likely to adopt smart device technology).^14^ System improvements for ease-of-use and customizability might increase provider use and limit the potential for user frustration or overuse; too many notifications would likely prove counterproductive to ED flow.

The overall magnitude of improvements observed in our study is similar to a trial of smartphone, troponin-result push notifications, in which Verma et al. found a 26-minute improvement in lag time from troponin result to patient disposition.^11^ Our institution almost exclusively uses a point-of-care troponin test in the ED and thus we could not study the specific clinical scenario of troponin to disposition in our study. A study of radiologic critical test results reported via text message to physicians similarly showed improved response time in ED patients.^15^

Our study specifically examined clinical scenarios in which the authors felt knowledge of a test result would most clearly lead to either a disposition decision or an additional test order, and hence a measurable effect. These specific clinical situations represent only a small proportion of the total ED volume and clinical caseload during the study period. While prolonged length of stay and ED crowding are multifactorial in etiology and the lag time between result availability and physician action is a small contributor,^8^ these results suggest that push notifications were potentially effective in modestly decreasing time to decision-making for providers opting-in for push notifications. This is also likely true in more complex clinical situations that were not studied. Further investigation is needed to identify and measure the impact of push notifications more broadly in the ED.

**Table:** 

Clinical scenario	Number of studies		Median minutes (IQR)		p value

	No Notifications Cohort	Notification Cohort	No Notifications Cohort (IQR)	Notification Cohort (IQR)	
		
CXR result to disposition	31592	516	80 (32–162)	56 (18–141)	p<0.01
BMP result to disposition	82946	354	128 (62–225)	116(33–226)	p<0.01
UA result to disposition	39510	397	105 (43–200)	55 (16–144)	p<0.01
RPP result to disposition	2991	168	80 (28–181)	37 (10–116)	p<0.01
D-dimer result to CTPA order	863	35	14 (6–30)	6 (2.5–17.5)	p<0.01
Hb result to transfusion order	852	18	36 (15–83)	17 (11–118)	p=0.73

IQR, interquartile range; CXR, chest radiograph; BMP, basic metabolic panel; UA, urinalysis; RPP, respiratory pathogen panel; CTPA, computed tomography pulmonary angiography; Hb, hemoglobin.

## LIMITATIONS

There are multiple limitations to this study. Our retrospective study was only able to show a correlation between result push notifications and improved time to decision-making. In addition, because each provider independently made the decision on whether or not to subscribe to a given test push notification, there may have been a potential selection bias: it is possible that providers who were motivated to subscribe to such alerts may also be those who are more efficient in general. It’s also possible providers subscribed to push notifications more often in situations in which they could quickly disposition a patient pending that single result.

The admission rate for the notification cohort was slightly higher than that for the encounters without a push notification. It is possible that slight differences in patient characteristics between the two groups may explain part of the difference in efficiency. We attempted to limit this shortcoming by also studying two scenarios in which patient factors would not affect efficiency (time to CTPA and time to transfusion).

The study only examined six different test results and clinical scenarios. It is possible that we chose scenarios that showed an improvement, whereas tests not studied (eg, extremity radiograph results) may not have demonstrated an effect. The finding of decreased lag time in every scenario studied suggests that the efficiency observed is likely generalizable to other types of studies.

## CONCLUSION

Implementation of a push notification system for test result availability in the ED was associated with a decrease in lag time between test result and provider decision-making in several clinical scenarios. However, push notifications were used in only a minority of all ED patient encounters during the study period. The use of push notifications may play a role in improving the timeliness of care delivered in the ED.

## Figures and Tables

**Table 1:** Characteristics of the no push notification and push notification cohorts.

Characteristcs	No Notifications Cohort, n = 148,391	Notifications Cohort, n = 4,183
Age, median	41	51
Women (%)	50.6%	51.8%
Admission rate (%)	17.2%	21.9%
ESI*	3.87	3.35

*ESI*, Emergency Severity Index (lower values signify higher patient acuity).
